# Heat shock preconditioning mesenchymal stem cells attenuate acute lung injury via reducing NLRP3 inflammasome activation in macrophages

**DOI:** 10.1186/s13287-021-02328-3

**Published:** 2021-05-17

**Authors:** Haijin Lv, Xiaofeng Yuan, Jiebin Zhang, Tongyu Lu, Jia Yao, Jun Zheng, Jianye Cai, Jiaqi Xiao, Haitian Chen, Shujuan Xie, Ying Ruan, Yuling An, Xin Sui, Huimin Yi

**Affiliations:** 1 Department of Surgical Intensive Care Unit, the Third Affiliated Hospital of Sun Yat-sen University, Guangzhou, 510630 Guangdong Province China; 2 Guangdong Key Laboratory of Liver Disease Research, Key Laboratory of Liver Disease Biotherapy and Translational Medicine of Guangdong Higher Education Institutes, the Third Affiliated Hospital of Sun Yat-sen University, Guangzhou, 510630 China; 3 Department of General Intensive Care Unit, The Third Affiliated Hospital of Sun Yat-sen University, Guangzhou, 510630 China; 4 Department of Hepatic Surgery and Liver Transplantation Center, The Third Affiliated Hospital of Sun Yat-sen University, Guangzhou, 510630 China; 5 Vaccine Research Institute of Sun Yat-sen University, Biotherapy Center, The Third Affiliated Hospital of Sun Yat-sen University, Guangzhou, China; 6 Department of Thyroid and Breast Surgery, The Third Affiliated Hospital of Sun Yat-sen University, Guangzhou, 510630 China

**Keywords:** Heat shock, Umbilical cord-derived mesenchymal stem cells, Acute lung injury, Macrophage, NLRP3 inflammasome

## Abstract

**Objectives:**

Acute lung injury (ALI) remains a common cause of morbidity and mortality worldwide, and to date, there is no effective treatment for ALI. Previous studies have revealed that topical administration of mesenchymal stem cells (MSCs) can attenuate the pathological changes in experimental acute lung injury. Heat shock (HS) pretreatment has been identified as a method to enhance the survival and function of cells. The present study aimed to assess whether HS-pretreated MSCs could enhance immunomodulation and recovery from ALI.

**Materials and methods:**

HS pretreatment was performed at 42 °C for 1 h, and changes in biological characteristics and secretion functions were detected. In an in vivo mouse model of ALI, we intranasally administered pretreated umbilical cord-derived MSCs (UC-MSCs), confirmed their therapeutic effects, and detected the phenotypes of the macrophages in bronchoalveolar lavage fluid (BALF). To elucidate the underlying mechanisms, we cocultured pretreated UC-MSCs with macrophages in vitro, and the expression levels of inflammasome-related proteins in the macrophages were assessed.

**Results:**

The data showed that UC-MSCs did not exhibit significant changes in viability or biological characteristics after HS pretreatment. The administration of HS-pretreated UC-MSCs to the ALI model improved the pathological changes and lung damage-related indexes, reduced the proinflammatory cytokine levels, and modulated the M1/M2 macrophage balance. Mechanistically, both the in vivo and in vitro studies demonstrated that HS pretreatment enhanced the protein level of HSP70 in UC-MSCs, which negatively modulated NLR family pyrin domain containing 3 (NLRP3) inflammasome activation in alveolar macrophages. These effects were partially reversed by knocking down HSP70 expression.

**Conclusion:**

HS pretreatment can enhance the beneficial effects of UC-MSCs in inhibiting NLRP3 inflammasome activation in macrophages during ALI. The mechanism may be related to the upregulated expression of HSP70.

**Graphical abstract:**

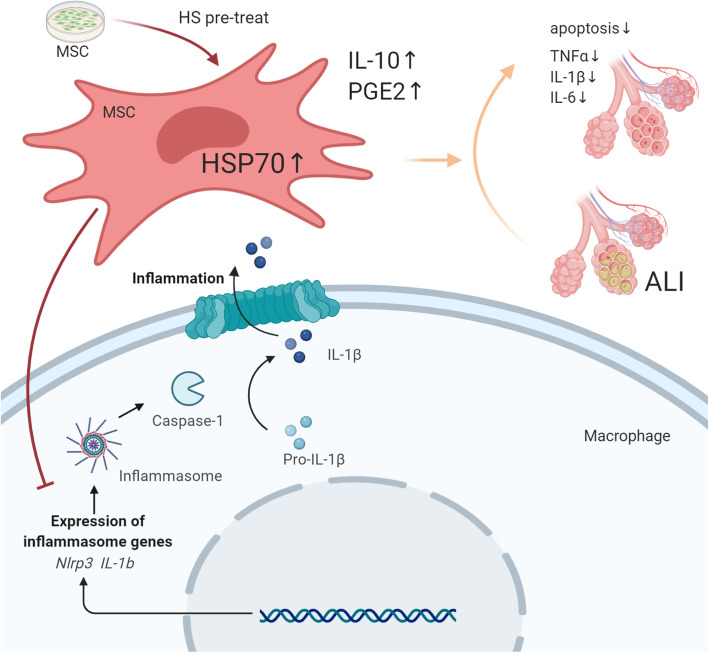

**Supplementary Information:**

The online version contains supplementary material available at 10.1186/s13287-021-02328-3.

## Introduction

Acute lung injury (ALI) is a common inflammatory disease that is pathologically characterized by diffuse inflammation and subsequently increased vascular permeability in the lung, which lead to reduced alveolar gas exchange and ultimately to the development of acute respiratory distress syndrome (ARDS) [1, 2]. ARDS remains a common and life-threatening lung disease, and the associated mortality rate ranges from 30 to 50% [3, 4]. Although advances in health care, including extracorporeal high-frequency oscillatory ventilation and carbon dioxide removal, have been made, there is still no pharmacological approach that is effective for the treatment of ARDS [5]. Therefore, the exploration of promising therapeutic strategies for targeting these pathophysiological features to alleviate ARDS is urgently needed.

As an imbalance between anti-inflammatory and proinflammatory factors is one of the major characteristics of ARDS, cytokine modulation may become a potential treatment approach [6]. Alveolar macrophages, which are the main components of the lung immune system and are located at the air-tissue interface, play heterogeneous roles in lung injury, as their various functions depend on their phenotypes and the cytokines they secrete [7, 8]. In a murine model of ALI, proinflammatory macrophages (M1-polarized) predominately reside in the lung and induce pulmonary injury; however, these cells are repolarized and express surface markers associated with a reparative phenotype (M2-polarized) during the resolution of ALI [9]. Treatment with mesenchymal stem cells (MSCs) might be an ideal therapeutic approach because of their potential to contribute to immunoregulation and tissue repair. Our previous studies yielded reliable results on the beneficial effects of MSCs on ameliorating liver ischemia/reperfusion injury (IRI) in vivo and in vitro [10, 11]. In addition, we and others have also shown that MSCs can alleviate lipopolysaccharide (LPS)-induced ALI via their well-defined role in immunomodulation and their ability to differentiate into multiple different cell types, including adipocytes, osteoblasts and lung-related cell types [12–17].

Indeed, recent studies have focused on how to enhance the therapeutic effects of MSCs in various diseases. In a previous study, we demonstrated that preconditioning MSCs with rapamycin could increase their hepatoprotective effects against liver IRI by enhancing their immunosuppressive function and strengthening their migratory capacity [18]. Heat shock (HS) has also been identified as an effective method by which to increase cell survival and enhance cell function in vitro in various culture environments [19, 20]. Several studies reported that HS pretreatment of transfused MSCs enhanced their survival following administration to liver IRI and chemotherapy-induced granulosa cell apoptosis models [21, 22]. Other findings have revealed that HS pretreatment of transplanted cells could strengthen their therapeutic effects [23]. However, whether HS pretreatment enhances the ability of MSCs to protect against ALI is still unknown.

The aim of this study was to investigate the protective effects of HS-pretreated umbilical cord-derived MSCs (UC-MSCs) in alleviating ALI and the potential underlying mechanisms. We hypothesized that HS not only maintains the viability of MSCs but also enhances the pulmonary protective effects of MSCs during ALI by modulating the NLRP3 inflammasome in macrophages.

## Methods and materials

### Isolation and culture of UC-MSCs

The UC-MSC isolation procedures were conducted according to the Declaration of Helsinki and were approved by the Research Ethics Committee of the Third Affiliated Hospital of Sun Yat-sen University (Guangzhou, China). All these procedures were performed under aseptic conditions according to previously described methods [24]. After obtaining informed consent from the patients, fresh umbilical cords (UCs) were obtained and immersed in phosphate-buffered saline (PBS) at 4 °C. After removing the remnant blood by washing with PBS, the UCs were cut into 10-mm^3^ pieces and placed in type I collagenase (Sigma, USA) with 0.1% hyaluronidase and 3 mM CaCl_2_ and digested at 37 °C for 4 h. The samples were incubated in low-glucose Dulbecco’s modified Eagle’s medium (DMEM, 1 g/L, Gibco, Life, Australia) with 10% fetal bovine serum (FBS, PAN-Biotech, Germany) at 37 °C in 5% CO_2_ in a humidified atmosphere. The medium was replaced every 3 days to remove the nonadherent cells, while the remaining adherent cells were cultured and passaged.

### Preparation of HS-pretreated UC-MSCs

The procedure for preparing HS-pretreated UC-MSCs was performed as previously described [25]. In brief, UC-MSCs in the 3rd passage were collected, and the culture medium was replaced to remove the nonadherent cells. Then, the cells were exposed to HS conditions for 1 h in a 42 °C water bath and subsequently cultured at 37 °C in 5% CO_2_ in a humidified atmosphere for 48 h. The control group included UC-MSCs incubated under typical conditions without HS pretreatment (Fig. 2a).

### Cytotoxicity assay

CCK-8 assay: UC-MSCs were plated in 96-well plates at a density of 2000 cells/well. A Cell Counting Kit-8 (CCK-8, Kaiji Bio-Technology Co. Ltd., Jiangsu, China) was conducted after 12, 24, and 48 h of treatment according to the manufacturer’s protocol. The optical density (OD) was measured at 450 nm using an automatic microplate reader (Biotek Vermont, USA). PI/Annexin V: UC-MSCs were stained with Annexin V/propidium iodide to quantify cell apoptosis in each group following the manufacturer’s instructions. In brief, UC-MSCs were cultured in 6-well plates, treated according to the experimental design for each group for 48 h, harvested, and stained with Annexin V/PI (Kaiji Bio-Technology) for 15 min. The apoptosis rate was detected by flow cytometry.

### Multipotential differentiation of UC-MSCs

To detect the adipogenic and osteogenic differentiation potential of the cells, UC-MSCs were incubated with media that specifically induced adipogenesis and osteogenesis (Gibco, Life Technologies, NY, USA), respectively. The medium of each specimen was replaced every 3 days. The ability of the UC-MSCs to differentiate into adipocytes and osteocytes was evaluated by Oil Red O and Alizarin Red S, respectively, after 14 or 21 days of incubation. For chondrogenic differentiation, 500,000 cells were pelleted in 15 mL conical tubes via centrifugation at 400*g* for 10 min. After 48 h in the appropriate basal medium for the cell type, chondrogenesis was induced with chondrogenic media containing of DMEM, as described previously. Pellets were maintained in culture for 28 days, and fixed in a 10% formalin solution prior to paraffin embedding and sectioning. Pellets were then stained with toluidine blue.

### Flow cytometry analysis

The cell surface antigens of the UC-MSCs were detected using flow cytometry analysis. After reaching 70–80% confluence, the cultured cells were trypsinized and incubated with monoclonal antibodies, including PE-Cy7-CD34, PE-CD29, PE-Cy7-CD45, APC-CD44, APC-CD90, FITC-CD105, and FITC-CD73, for 30 min at 4 °C in the dark. Flow cytometry analysis was performed using an eight-color FACSCalibur™ flow cytometer (BD Biosciences, NJ, USA).

We also used flow cytometry analysis to detect changes in macrophage polarization in vivo and in vitro. For surface marker staining, cells were harvested from both bronchoalveolar lavage fluid (BALF) and in vitro experiments and were incubated with primary APC-CD206 and PE-CD11b antibodies at 4 °C for 30 min in the dark. The fluorescence of the cells was evaluated using an eight-color FACSCalibur™ flow cytometer.

All the data were analyzed using FlowJo 7.6 software (Becton Dickinson, USA).

### Quantitative real-time reverse transcriptase-polymerase chain reaction

Total RNA extraction and quantitative real-time reverse transcriptase-polymerase chain reaction (qRT-PCR) were conducted following a previous report [18]. Total RNA was extracted from UC-MSCs and macrophages using TRIzol (Invitrogen) according to the manufacturer’s protocol. A Biophotometer Plus (Eppendorf, Germany) was used to detect the amount and purity of the total RNA at absorbance wavelengths of 260 nm and 280 nm. Then, the RNA was reverse transcribed into cDNA by a Transcriptor First-Strand cDNA Synthesis Kit (Roche, Applied Science, USA), and the cDNA was amplified for 10 min at 65 °C, incubated at 55 °C for 30 min, deactivated at 85 °C for 5 min, and finally stored at 4 °C for 5 min in a PCR thermal cycler (Bio-Rad, USA). RT-qPCR was conducted with SYBR Master Mix (Roche Applied Science) using a reverse transcription system (LC-480, Roche, USA). β-actin was used as the housekeeping gene. The sequences of the target gene-specific primers used in this study are shown in Supplementary Table 1.

### Enzyme-linked immunosorbent assay

Enzyme-linked immunosorbent assay (ELISA) was performed as previous published criteria [26]. The levels of IL-10, PEG2, and TGF-β in the MSC-conditioned medium (MSC-CM) and the concentrations of TNF-α, IL-1β, and IL-6 in the supernatants and BALF were measured using ELISA kits following the manufacturer’s instructions (EIAAB Science Company, Wuhan, China). An automatic microplate reader (Biotek, Vermont, USA) was used to detect the optical density (OD) at 450 nm.

### Immunoblotting

Lung tissues and macrophages were lysed with cold radioimmunoprecipitation assay (RIPA) buffer containing 50 mM Tris-HCl (pH 7.4), 150 mM NaCl, 0.1% Triton X-100, 10% SDS, 10% sodium deoxycholate, 2 mM EDTA, and a protease inhibitor cocktail (Roche, Basel, Switzerland). Equal amounts of proteins were subjected to 12% SDS polyacrylamide gel electrophoresis (PAGE) and subsequently transferred onto polyvinylidene difluoride (PVDF) membranes (Millipore, Billerica, MA, USA). After incubation in 5% nonfat milk for 1 h to block nonspecific antigens, the membranes were incubated with primary antibodies, including antibodies against NLRP3, ACS, pro-Caspase 1, cleaved Caspase 1, and IL-1 β, at 4 °C overnight. After washing three times with tris-buffered saline with tween (TBST), the membranes were incubated with the secondary antibodies (1:5000, Sigma-Aldrich) for 1 h at room temperature. Then, the membranes were incubated with an enhanced chemiluminescence (ECL) substrate, and the blots were visualized using a FluorChem System imager (ProteinSimple, CA, USA). ImageJ software (USA) was used to analyze the intensities of the bands.

### Animals

C57BL/6 mice (B6 mice, male, 8–10 weeks, 22–25 g) were purchased from Biomedical Research Institute of Nanjing University (Jiangsu, China). All the mice were housed in the Laboratory Animal Center of South China Agricultural University under specific pathogen-free (SPF) conditions and were cared for according to the Guideline of South China Agricultural University for Animal Experimentation. The animals were maintained in an environment with a constant temperature (20 °C), humidity (50%), and dark and light cycle (12 h) and received free access to a standard laboratory diet and water.

### LPS-induced ALI model and BALF collection

All the animal experiments were conducted according to the guidelines of the Ethics Committee on Animal Welfare, South China Agricultural University. The standard procedures for establishing a mouse model of LPS-induced ALI were performed as previously described [27]. In brief, the mice were anesthetized by intraperitoneal injection of pentobarbital sodium (0.1%, 100 μl/10 g) and then divided into four groups: PBS + PBS, lipopolysaccharide (LPS) + PBS, LPS + UC-MSCs, and LPS + HS-UC-MSCs. The mouse model of ALI was established by intranasally dripping LPS (5 mg/kg, Sigma, USA). The sham group was treated with the same volume of PBS. After 4 h of treatment, the mice were intranasally administered UC-MSCs (1 × 10^4^/30 μl), PBS (30 μl) or HS-UC-MSCs (1 × 10^4^/30 μl) according to the experimental design of each group mentioned above. The mice in the four groups were euthanized 24 h after model establishment.

To collect the BALF, an intravenous infusion needle (BD, USA) was used to flush the lung 3 times with 0.5 ml of PBS prechilled at 4 °C through the trachea. After centrifugation at 500*g* for 10 min at 4 °C, the supernatant of the BALF was removed, and the cells were resuspended in ice-cold PBS. The total number of cells was counted using a hemocytometer, and the number of neutrophils was counted after Wright-Giemsa staining.

### Lung wet/dry weight ratio evaluation

The lung wet/dry weight ratio was calculated to evaluate the severity of lung edema. In brief, lung tissues were obtained immediately after the mice were sacrificed, and the wet weight value was recorded. Then, the samples were placed in a 65 °C incubator for 72 h, and the dry weight value was recorded.

### Lung histopathology and injury scoring

Four-micrometer-thick paraffin-embedded lung tissue sections were prepared and stained with hematoxylin and eosin (H&E). The histological scores, which indicated the severity of lung injury, were determined according to a previous study; this score was calculated after assessing hyaline membrane formation, alveolar and interstitial inflammation and hemorrhage, necrosis, atelectasis, and edema [28]. The slides were evaluated under a light microscope by an observer who did not know the experimental design of the groups. Five fields were selected from each section to calculate the injury score. Lung injury scores were quantified by an investigator blinded to the treatment groups using published criteria, which gives an overall score of between 0 and 1 [29].

### Fluorescent TUNEL staining

To investigate cell apoptosis in the lung, we performed a fluorescent TUNEL (terminal deoxynucleotidyl transferase-mediated dUTP nick end labeling) assay using a commercial in situ apoptosis detection kit (Roche Diagnostics, Indianapolis, IN, USA) on 16-μm-thick cryosections. Briefly, after washing with equilibration buffer, the cryosections were incubated with the TUNEL reaction mixture, including the enzyme and label solution, for 60 min in the dark at 37 °C. Then, the sections were treated with DAPI for 2 min in the dark and mounted using Prolong Diamond Antifade Mounting Agent. The slides were investigated under a fluorescent inverted microscope (Leica, Germany).

### Cell coculture experiments

UC-MSCs or HS-pretreated UC-MSCs were seeded on a Transwell insert (0.4 μm, Corning) at a density of 4 × 10^5^ cells/ml. Human THP-1 monocytic cells (ATCC, Manassas, VA, USA) were cultured in T-75 flasks in RPMI 1640 (Sigma-Aldrich, St. Louis, MO, USA) containing 10% FBS, 1% penicillin/streptomycin, and 50 μM β-mercaptoethanol (Sigma-Aldrich, St. Louis, MO, USA) incubating at 37 °C in a 95% humidified atmosphere and 5% CO_2_. Cells were subcultured when they reached a concentration of 8 × 10^5^ cells/ml. In addition, the THP-1 macrophage cell line was plated in the lower chamber of a 6-well plate at a concentration of 5 × 10^5^ cells/ml and stimulated with PMA (50 ng/ml) for 24 h. Then, the upper chamber containing cultured UC-MSCs and HS-UC-MSCs was placed in the 6-well plate, cocultured with the THP-1 cells, and treated with LPS (1 μg/ml) and IFN-γ (50 ng/ml) for 24 h [30]. Finally, the THP-1 cells were harvested using EDTA (0.5 mM).

### Statistical analysis

The data are presented as the mean ± standard error of the mean (SEM) or as direct values. As appropriate, differences between groups were compared using unpaired two-tailed Student’s *t* test or one-way analysis of variance (ANOVA). A probability value (*p* value) < 0.05 was considered statistically significant. The statistical analyses were conducted using GraphPad Prism 7 software (GraphPad Software, San Diego, CA).

## Results

### Culture and identification of UC-MSCs

**Fig. 1:**
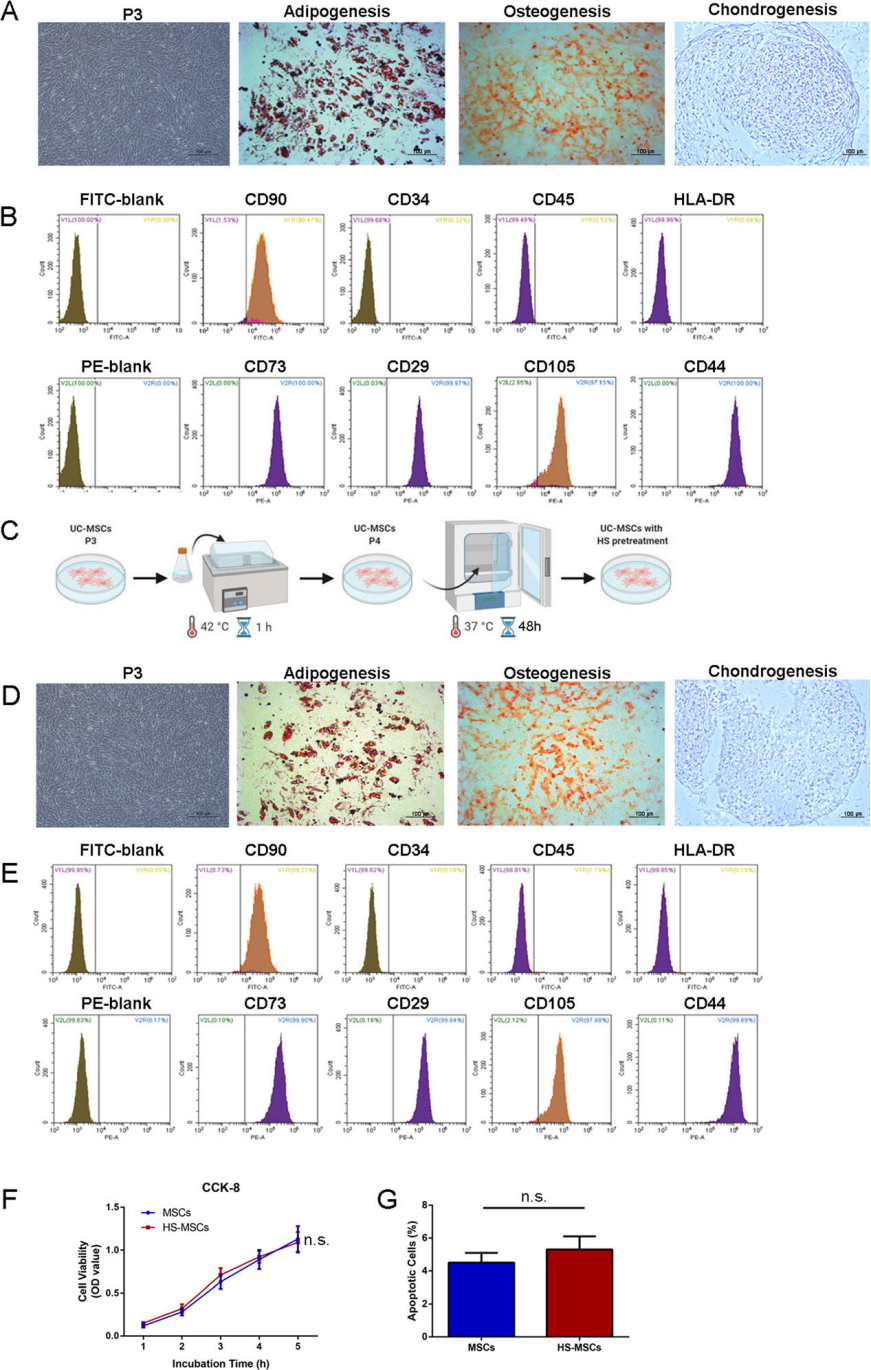
The optimal condition of HS pretreatment did not affect the viability and biological characteristics of UC-MSCs. **a** The fibroblast-like morphology and multi-differentiation potential of cultured UC-MSCs. Oil red O staining of UC-MSCs cultured in medium that specifically induced adipogenesis 14 days. Alizarin Red solution staining of UC-MSCs cultured in medium that specifically induced osteogenesis for 21 days. UC-MSC pellets induced chondrogenesis 28 days stained with toluidine blue (scale bar = 100 μm). **b** Flow cytometry analysis of the surface markers of UC-MSCs. **c** The procedure for obtaining HS-pretreated UC-MSCs. **d** Representative images of HS-pretreated UC-MSCs, which differentiated via adipogenesis, osteogenesis, and chondrogenesis (scale bar = 100 μm). **e** Flow cytometry analysis of the surface markers of HS-pretreated UC-MSCs. **f** UC-MSCs and HS-pretreated UC-MSCs were subjected to a CCK-8 assay after 0, 1, and 2 days of culture to assess cellular viability. **g** The apoptosis rate of each group was detected using Annexin V/propidium iodide staining. The data are presented as the mean ± SEM. ns, not significant (all *p* values were obtained by unpaired two-tailed Student’s *t* test)

As shown in Fig. 1a, the UC-MSCs were identified as a population of fibroblast-like cells. After incubation for 14 days, 21 days, or 28 days in specific media, the cells differentiated into adipocytes, osteocytes, or chondrocytes, as shown by staining with Oil red O, Alizarin Red solution, and toluidine blue, respectively. Examination of their cell surfaces showed positive expression of CD73, CD90, CD29, CD105, and CD44 and negative expression of CD34, CD45, and HLA-DR (Fig. 1b).

### The biological features and viability of HS-pretreated UC-MSCs

As shown in Fig. 1c, pretreatment with HS for 1 h and subsequent culture under typical conditions for 48 h did not affect the morphology, the multi-differentiation potential (Fig. 1d), or surface characteristics of the UC-MSCs (Fig. 1e). The CCK-8 assay showed that there was no significant difference in the cell proliferation rate between the UC-MSCs cultured under typical conditions and the HS-pretreated UC-MSCs (Fig. 1f). The flow cytometry results revealed that the apoptotic rate of the HS-pretreated UC-MSCs was not significantly different from that of the control cells (Fig. 1g).

### Administration of HS-pretreated UC-MSCs attenuates LPS-induced ALI

**Fig. 2:**
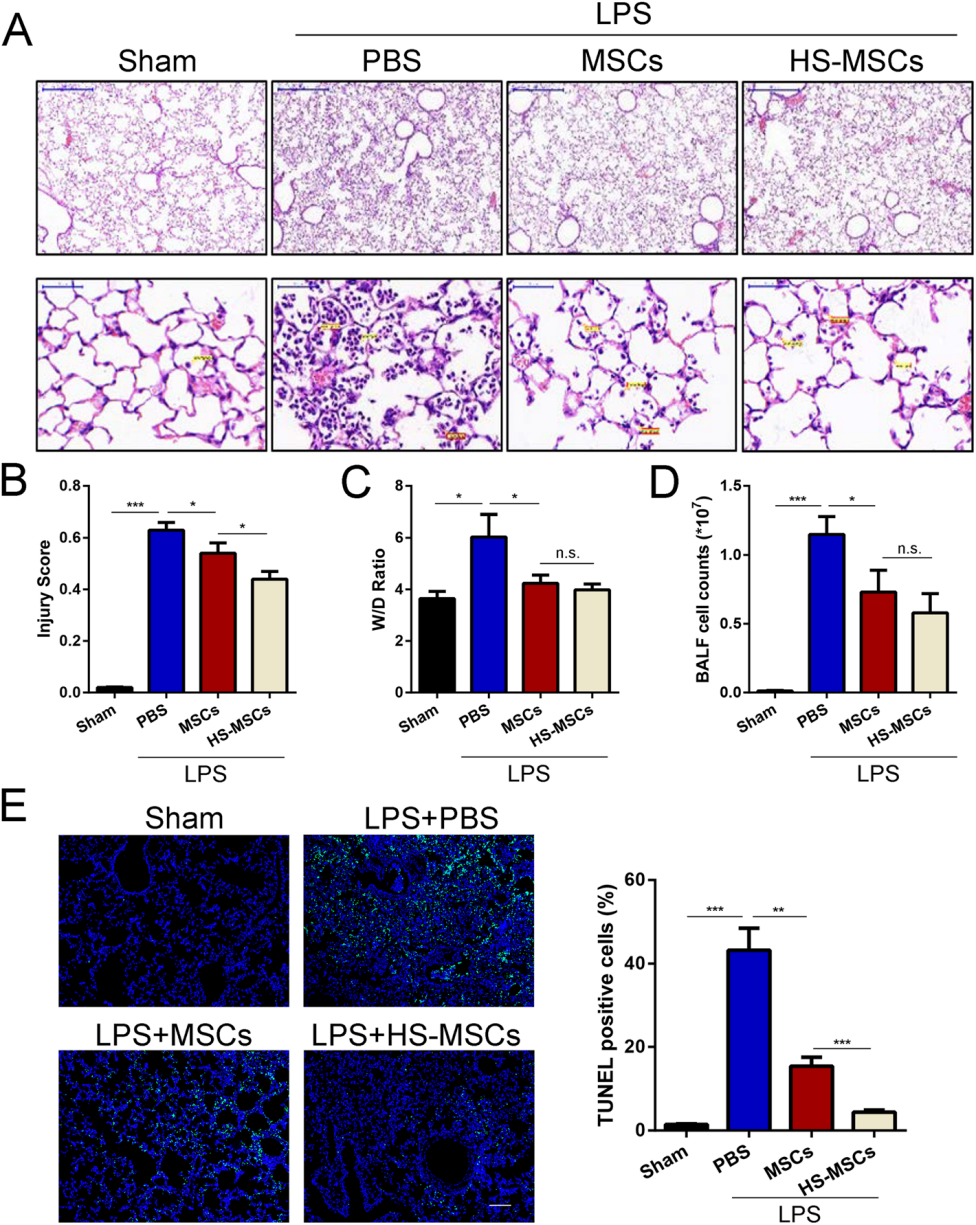
HS-pretreated UC-MSCs attenuate LPS-induced ALI. **a** Hematoxylin and eosin staining of lung tissues in each group to assess the amount of lung damage after ALI. Scale bar 200 μm. **b** The lung injury score of each group was calculated by selecting five fields in each section. The results are presented as the mean ± SEM (*n* = 5 mice/group). **c** The wet/dry ratio of lung tissue of each group was obtained to evaluate lung edema. The results are presented as the mean ± SEM (*n* = 5 mice/group). **d** The total cell count and neutrophil count in the BALF from each group were examined at 24 h after LPS treatment to assess the inflammatory response. The results are presented as the mean ± SEM (*n* = 5 mice/group). **e** Representative images of TUNEL staining of lung sections from each group were collected. Scale bar 200 μm. The data are presented as the mean ± SEM (*n* = 5 mice/group). *p < 0.05, **p < 0.01, ***p < 0.001 (all *p* values were obtained by one-way ANOVA)

We assessed the severity of lung injury in each group to determine whether HS-pretreated UC-MSCs can improve pulmonary function after LPS administration. A mouse model of ALI was established by the intratracheal injection of LPS. After 24 h, lung injury was pathologically confirmed and was characterized by lung edema, widespread septal thickening, and neutrophil/mononuclear infiltration into alveolar spaces; however, these changes were significantly improved after the administration of UC-MSCs, and treatment with HS-pretreated UC-MSCs led to the enhanced recovery of these histopathological changes (Fig. 2a). The lung injury score, which was evaluated based on H&E staining of lung sections, showed consistent results (Fig. 2b). Moreover, the results showed that LPS induced an increase in the lung wet/dry weight ratio in the mice, while UC-MSCs significantly ameliorated pulmonary edema (Fig. 2c). Interestingly, a better therapeutic effect was observed in the HS-pretreated UC-MSC group than in the UC-MSC group (Fig. 2c). Exchange in BALF is an important indicator for assessing lung function. The results from cell smears showed that HS pretreatment could further enhance the UC-MSC-induced decrease in the BALF inflammatory cell count (Fig. 2d). In addition, a TUNEL assay was performed to evaluate endothelial apoptosis in each group. As shown in Fig. 2e, the apoptotic index of the lung endothelium obviously increased after exposure to LPS, whereas the increased index was markedly decreased after treatment with UC-MSCs. Moreover, in mice administered HS-pretreated UC-MSCs, the apoptotic index of the lung endothelium was significantly lower than that in the mice administered UC-MSCs. These results demonstrated that HS pretreatment plays a critical role in enhancing the lung-protective effects of UC-MSCs.

### HS-pretreated UC-MSCs modulate the functions of alveolar macrophages in vivo and in vitro

**Fig. 3:**
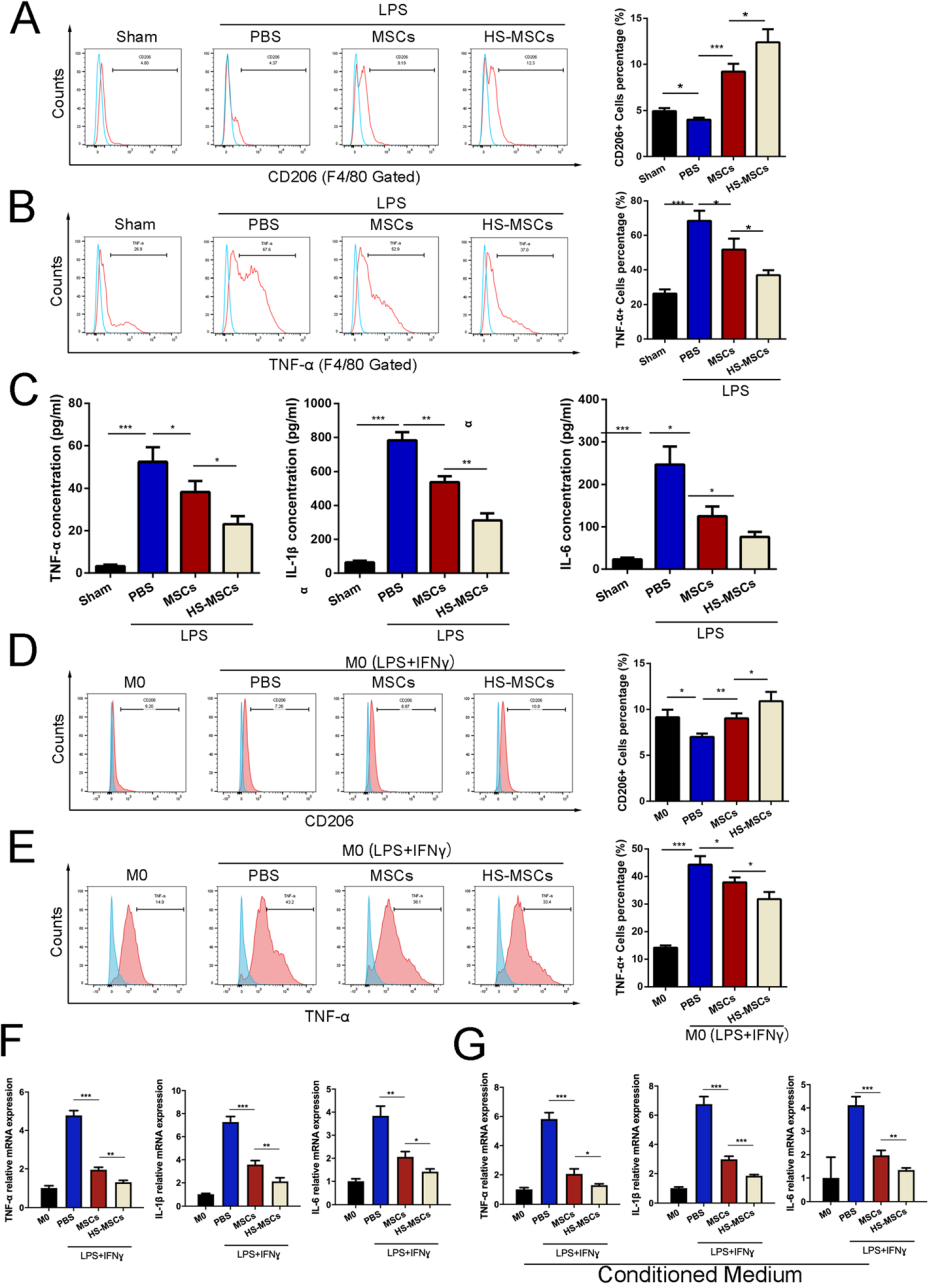
HS-pretreated UC-MSCs modulate the phenotypic polarization and secretion function of alveolar macrophages. The surface markers CD206 (**a**) and TNF-α (**b**) of alveolar macrophages from BALF were detected by flow cytometry analysis. The data are presented as the mean ± SEM (*n* = 5 mice/group). **c** The levels of TNF-α, IL-1β, and IL-6 in BALF from each group were measured by ELISA. The data are presented as the mean ± SEM (*n* = 5 mice/group). Flow cytometry analyses of CD206 (**d**) and TNF-α (**e**) expression in macrophages in each group. **f** The mRNA expression of TNF-α, IL-1β, and IL-6 in THP-1 cells co-cultured with each group of MSCs. **g** The mRNA expression of TNF-α, IL-1β, and IL-6 in THP-1 cells treated with conditioned medium from each group of MSCs. The data are presented as the mean ± SEM (*n* = 3/group). *p < 0.05, **p < 0.01, ***p < 0.001 (all *p* values were obtained by one-way ANOVA)

Increasing evidence has demonstrated that the polarization of alveolar macrophages is a critical factor that affects the pathological process of ALI [31]. Thus, we next collected BALF and detected changes in the alveolar macrophage phenotypes in each group. The flow cytometry results showed that UC-MSCs promoted the expression of the anti-inflammatory marker CD206 (Fig. 3a) and decreased the LPS-induced increased expression of the proinflammatory marker TNF-α in alveolar macrophages (F4/80+) (Fig. 3b). Compared with UC-MSCs cultured under typical conditions, UC-MSCs pretreated with HS exhibited an enhanced immunoregulatory effect in inducing M2 macrophage polarization (Fig. 3a, b). Furthermore, we detected the concentrations of several cytokines to indirectly evaluate the secretion function of alveolar macrophages. As shown in Fig. 3c, LPS promoted the secretion of various cytokines, including TNF-α, IL-1β, and IL-6, from macrophages, and UC-MSCs reversed these changes in macrophage secretion. As expected, HS pretreatment further enhanced these immunoregulatory effects of UC-MSCs. We further used LPS-stimulated THP-1 cells (macrophage cell line) as an in vitro model and also detected the levels of M2 markers in each group. After stimulation, the expression of CD206 was significantly increased in macrophages cocultured with non-pretreated UC-MSCs compared to control macrophages, and this effect was further enhanced in macrophages after coculture with HS-pretreated UC-MSCs (Fig. 3d). The levels of M1 markers were significantly increased after treatment with LPS compared with the control, while coculture with non-pretreated or HS-pretreated UC-MSCs significantly reversed this effect (Fig. 3e). In model of co-cultured with MSCs, we detected the mRNA level of inflammatory cytokines mRNA expression in THP-1. The results showed that the HS-MSC could further decrease the mRNA level of TNF-α, IL-1β, and IL-6 in THP-1 cells (Fig. 3f). Meanwhile, to detect whether LPS-preconditioned MSCs also have some cytoprotective effects, we collected the conditioned medium (CM) from HS-MSCs to treat THP-1 and analyze the inflammatory cytokines mRNA expression by RT-qPCR. The results showed similar with co-cultured experiments (Fig. 3g). These results suggested that HS pretreatment enhances the effects of UC-MSCs in modulating the polarization and secretion functions of alveolar macrophages.

### HS-pretreated UC-MSCs reduce the activation of the NLRP3 inflammasome in macrophages

**Fig. 4:**
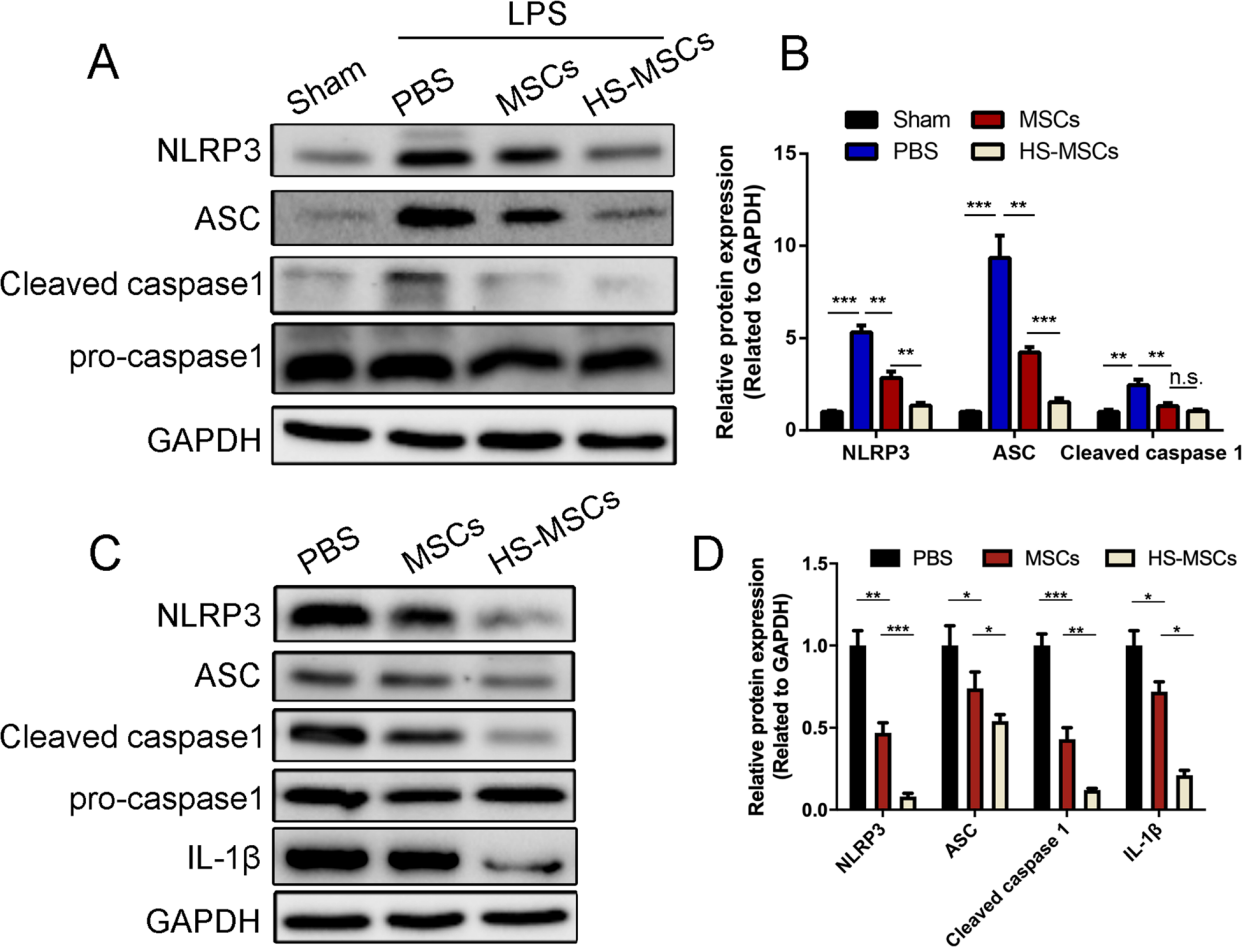
HS-pretreated UC-MSCs reduce NLRP3 inflammasome activation in lungs and alveolar macrophages. **a** Protein levels of NLRP3, ASC, pro-Caspase 1, and cleaved-Caspase 1 in murine lung samples were detected by immunoblotting. **b** The immunoblotting results were quantified by analyzing the gray values. The data are expressed as the mean ± SEM (*n* = 5 mice/group). **c** Protein levels of NLRP3, ASC, pro-Caspase 1, cleaved-Caspase 1, and IL-1 β in the macrophage cell line (THP-1 cells) were detected by immunoblotting. **b** The immunoblotting results were quantified by analyzing the gray values. The data are expressed as the mean ± SEM (*n* = 3/group). *p < 0.05, **p < 0.01, ***p < 0.001 (all *p* values were obtained by one-way ANOVA)

It is known that the expression of the components of the NLRP3 inflammasome is positively associated with the severity of ALI [32], and MSCs can significantly reduce NLRP3 inflammasome component expression. Therefore, we further investigated whether HS pretreatment enhanced the therapeutic effect of UC-MSCs on NLRP3 inflammasome activation. As shown in Fig. 4a, b, the increase in the protein expression of NLRP3, ASC, pro-Caspase 1, and cleaved-Caspase 1 in the lung tissues lysis solution after LPS exposure was markedly decreased after treatment with UC-MSCs, whereas the expression of these proteins in the HS-pretreated UC-MSC group was significantly lower than that in the UC-MSC group.

In previous studies, NLRP3 inflammasome components were demonstrated to be mainly expressed in activated macrophages. Thus, we also detected the effects of HS-pretreated UC-MSCs on NLRP3 inflammasome activation in a macrophage cell line (THP-1 cells) in vitro (Fig. 4c and d). The immunoblotting results showed that the increased NLRP3 activation in macrophages, as evidenced by the increased expression of NLRP3, ASC, pro-Caspase 1, cleaved-Caspase 1, and IL-1β, was obviously suppressed by UC-MSC treatment. Pretreatment with HS enhanced the effects of UC-MSCs in reducing NLRP3 inflammasome activation in macrophages. Together, these results indicate that HS pretreatment enhances the lung protective capacity of UC-MSCs in the ALI model by inhibiting the activation of the NLRP3 inflammasome in macrophages.

### HS pretreatment enhances the secretion abilities of UC-MSCs by increasing HSP70 expression

**Fig. 5:**
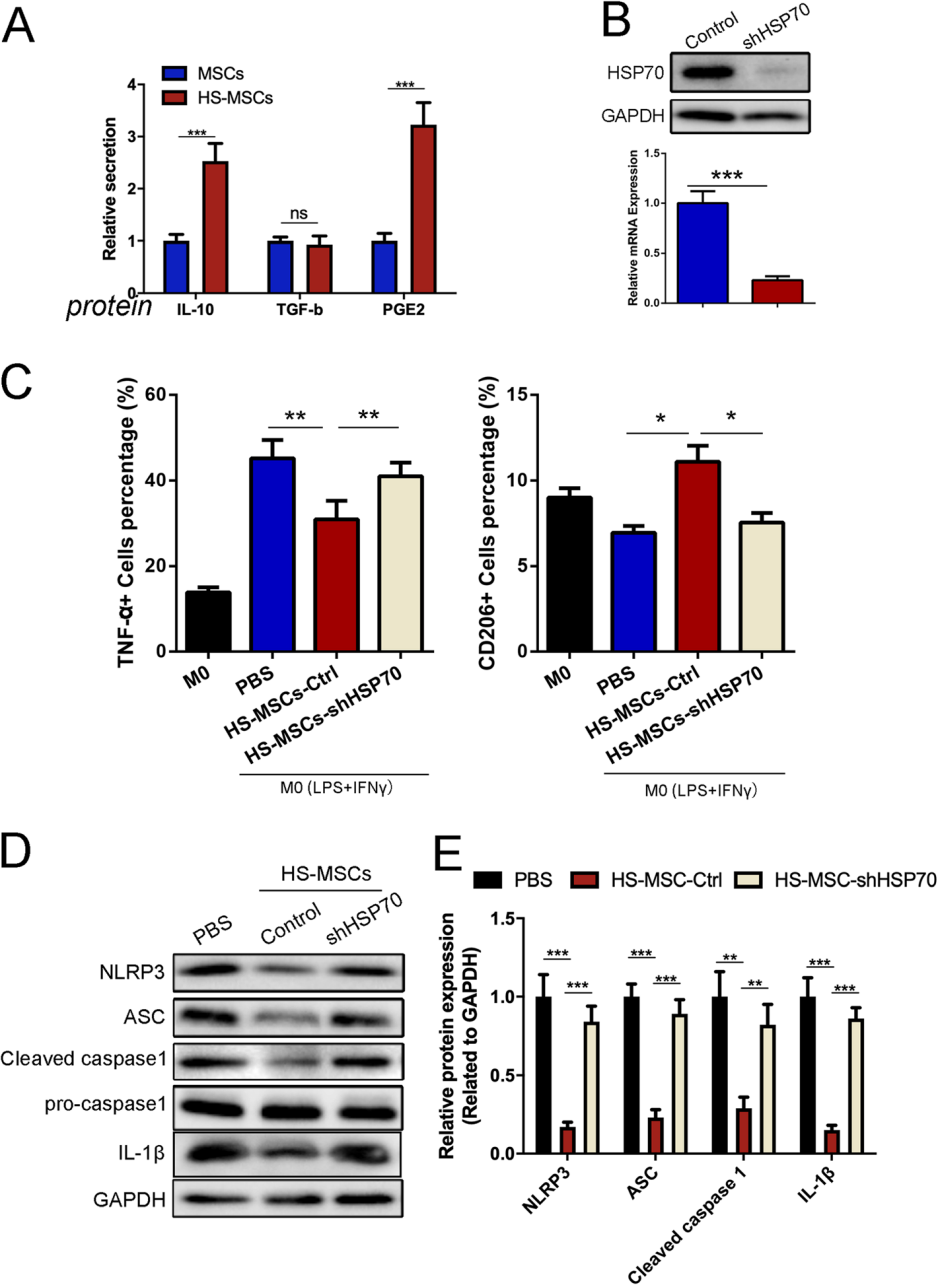
HS pretreatment enhanced the secretion abilities of UC-MSCs by increasing HSP70 expression. **a** The levels of IL-10, TGF-β, and PGE2 in UC-MSC culture supernatants from each group were measured by ELISA. The data are presented as the mean ± SEM (*n* = 3/group). **b** Protein levels of HSP70 in UC-MSCs after knockdown of HSP70 were detected by immunoblotting. The immunoblotting results were quantified by analyzing the gray values. The data are expressed as the mean ± SEM (*n* = 3/group). **c** Flow cytometry analyses of TNF-α and CD206 expression in macrophages of each group. The data are presented as the mean ± SEM (*n* = 3/group). **d** Protein levels of NLRP3, ASC, pro-Caspase 1, cleaved-Caspase 1, and IL-1β in macrophages were detected by immunoblotting. The immunoblotting results were quantified by analyzing the gray values. The data are expressed as the mean ± SEM (*n* = 3/group). *p < 0.05, **p < 0.01, ***p < 0.001 (all *p* values were obtained by one-way ANOVA)

We further explored the potential mechanism by which HS-pretreated UC-MSCs ameliorate ALI and reduce NLRP3 inflammasome activation in macrophages. In previous studies, both IL-10 and PGE2 were shown to negatively regulate inflammasome activation [33, 34]. Therefore, in this study, we detected the levels of IL-10, TGF-β, and PGE2 in culture supernatants to evaluate the secretion abilities of UC-MSCs in the presence or absence of HS. The ELISA results showed that compared with the control group, HS pretreatment caused UC-MSCs to synthesize and secrete notable levels of IL-10 and PGE2 but not TGF-β (Fig. 5a). In addition, as Fan et al. demonstrated that HSP70 promoted PGE2 production by regulating COX-2 [35, 36], we targeted the expression of HSP70 in the UC-MSCs in each group. To test the function of HSP70 in HS-pretreated UC-MSCs, HSP70 expression was reduced in MSCs using a lentivirus carrying a short hairpin RNA against HSP70 (shHSP70). The efficiency of HSP70 knockdown was confirmed by immunoblotting and RT-qPCR (Fig. 5b).

Compared to the HS-pretreated UC-MSCs, the shHSP70-expressing MSCs significantly rescued the decreased TNF-α secretion in the THP-1 macrophages. The expression of CD206 was significantly increased in the macrophages cocultured with HS-pretreated UC-MSCs compared to the control macrophages, and this change was reversed in macrophages cocultured with HS-pretreated shHSP70 MSCs (Fig. 5c). We also performed an immunoblotting assay to detect the inflammasome activation of the macrophages in each group. The results indicated that shHSP70 might reverse the effect of HS-pretreated UC-MSCs in reducing NLRP3 inflammasome activation in macrophages (Fig. 5d, e).

### Inhibition of HSP70 weakens the protective effect of HS-pretreated UC-MSCs in attenuating LPS-induced ALI

**Fig. 6:**
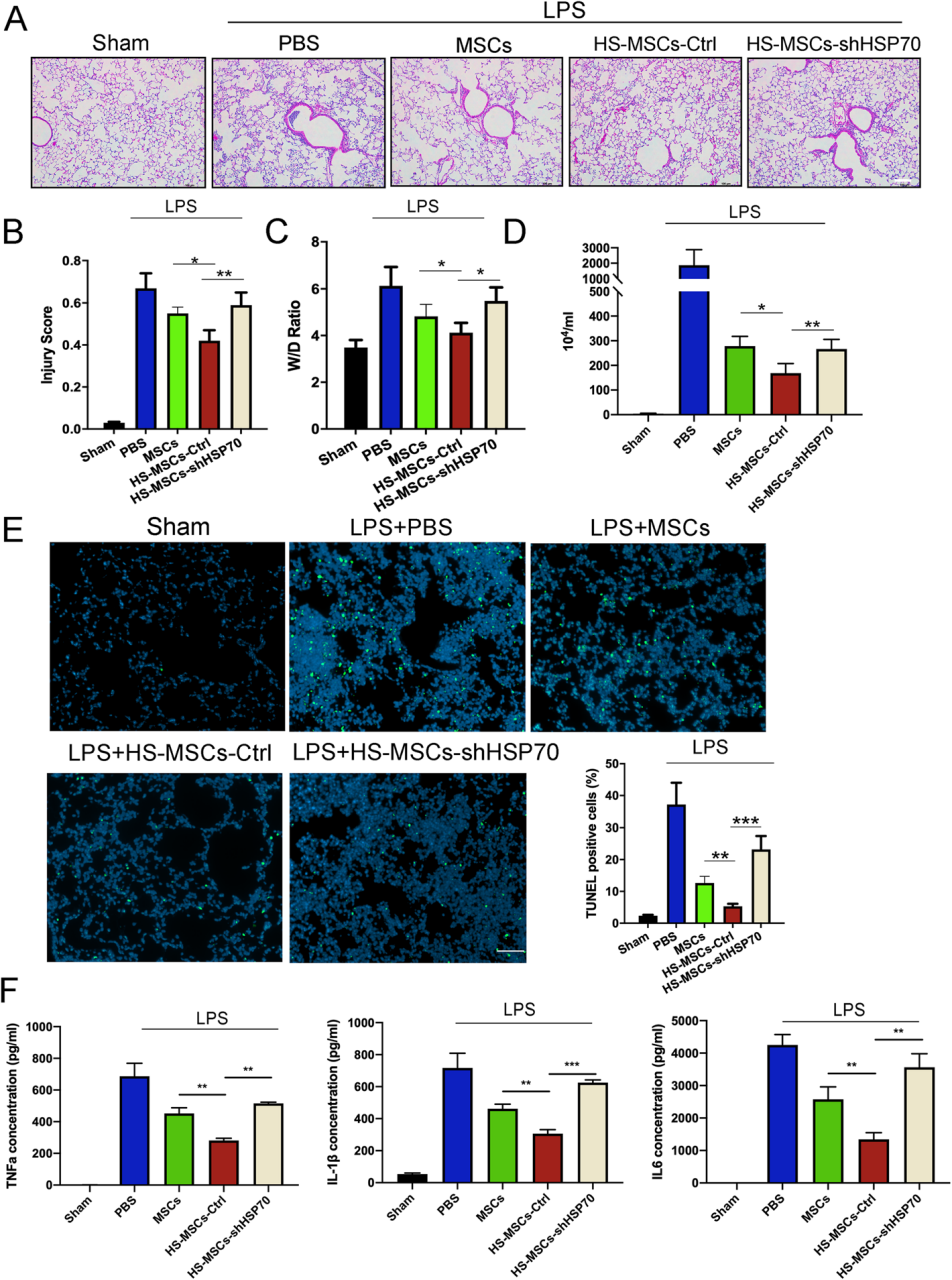
The protective effect of HS-pretreated UC-MSCs on attenuating LPS-induced ALI by regulating HSP70. **a** Hematoxylin and eosin staining of lung tissues from each group to assess the amount of lung damage after ALI. Scale bar 200 μm. **b** The lung injury score of each group was calculated by selecting five fields of each section. The results are presented as the mean ± SEM (*n* = 5 mice/group). **c** The wet/dry ratio of lung tissues from each group was obtained to evaluate lung edema. The results are presented as the mean ± SEM (*n* = 5 mice/group). **d** The total cell count and neutrophil count in the BALF from each group were examined at 24 h after LPS treatment to assess the inflammatory response. The results are presented as the mean ± SEM (*n* = 5 mice/group). **e** Representative images of TUNEL staining of lung sections from each group were collected. Scale bar 200 μm. The data are presented as the mean ± SEM (*n* = 5 mice/group). **f** The levels of TNF-α, IL-1β, and IL-6 in BALF from each group were measured by ELISA. The data are presented as the mean ± SEM (*n* = 5 mice/group). The data are expressed as the mean ± SEM (*n* = 3/group). *p < 0.05, **p < 0.01, ***p < 0.001 (all *p* values were obtained by one-way ANOVA)

Furthermore, we also evaluated the degree of lung injury in each group to explore whether HS pretreatment of UC-MSCs attenuates LPS-induced ALI by regulating HSP70. The results showed that HSP70 knockdown abolished the protective effect of HS-pretreated UC-MSCs, compared with NTC UC-MSC, as evidenced by the exacerbated pathological changes in the lung architecture (H&E staining and injury score) and the increased lung wet/dry weight ratio (Fig. 6a–c). In addition, HSP70 knockdown reversed the HS-pretreated UC-MSC-mediated reduction in BALF inflammatory cell counts (Fig. 6d). The results of investigating TUNEL-positive cells indicated that the role of HS-pretreated UC-MSCs in protecting alveolar epithelial cells was weakened by knocking down HSP70 (Fig. 6e). In addition, we also performed ELISAs to evaluate the inflammatory response, and the results showed that the significant decreases in proinflammatory cytokines, including TNF-α, IL-1β, and IL-6, induced by HS-pretreated UC-MSCs in this ALI model were markedly reversed after HSP70 knockdown (Fig. 6f).

## Discussion

To date, ALI remains one of the most serious respiratory inflammatory syndromes and is known to be a major factor affecting mortality. The current medical therapeutic approaches are not able to halt the progression of ALI [5]. Therefore, exploring optimal therapeutic strategies has been the subject of urgent investigation. With their immunomodulatory effects and ability to differentiate into a variety of cell types, MSCs have been considered a novel and effective therapeutic option for ALI. In a previous study, Lee et al. found that MSCs produce various soluble factors, including angiopoietin-1 (Ang-1), interleukin-10 (IL-10), keratinocyte growth factor (KGF), and prostaglandin E2 (PGE2), to improve lung epithelial and endothelial permeability, reduce inflammatory responses, enhance alveolar fluid clearance, and facilitate endothelial repair [37]. In addition, Khubutiya et al. demonstrated that these beneficial effects of MSCs on tissue repair mainly depended on their paracrine effects rather than differentiation ability [38]. Large-sample clinical trials evaluating the effects of MSCs on ALI are being conducted.

Recently, an increasing number of studies have focused on the strategies to enhance the therapeutic effectiveness of MSCs to widely promote the clinical administration of cellular therapy. Genetic modification is a particularly valuable approach because it directly integrates exogenous genes into cells to induce MSCs to produce desired proteins. To solve the problem of the low expression of homing receptors by MSCs, Yang et al. genetically overexpressed CXCR4 in MSCs using lentiviral vector transfection to enhance their homing efficiency and therapeutic effect in ALI [39]. Overexpressing IL-1 receptor-like-1 (ST2) could increase the anti-inflammatory effect of MSCs on ALI via the interleukin-33 (IL-33)/ST2 axis [40]. In addition, other studies focused on culturing MSCs with several specific cytokines or small molecule compounds to enhance their therapeutic effects. Witte et al. showed that prestimulation with inflammatory cytokines could enhance the immunomodulatory effects of UC-MSCs [41, 42]. Our previous study demonstrated that preconditioning UC-MSCs with rapamycin could increase cell migration and attenuate liver IRI via the CXCR4/CXCL12 axis [18]. In this study, we showed that heat shock (HS) pretreatment also enhanced the therapeutic potential of UC-MSCs during ALI. HS is a source of stress that facilitates organism and cell damage. However, recent studies showed that adaptive heat shock pretreatment could improve the viability and antiapoptotic properties of MSCs. Peng found that HS pretreatment effectively induced autophagy to decrease the apoptosis rate and increase the hepatoprotective effects of MSCs in liver IRI [21]. Other studies also reported that compared with MSCs, HS-pretreated MSCs were better suited to repairing chemotherapy-induced premature ovarian failure and preventing cisplatin-induced granulosa cell apoptosis [22, 25]. Inconsistent with the previous studies mentioned above, we directly selected HS conditions at 42 °C for 1 h according to Qing et al [22]. They proved that optimal HS condition was determined as 42 °C for 1 h and recovering for 48 h. The results of the biological characterization of the MSCs suggested that HS pretreatment did not affect the viability and antiapoptotic processes of the UC-MSCs. Furthermore, we established a mouse model of LPS-induced ALI, and the data indicated that the therapeutic effects of HS-pretreated UC-MSCs during ALI were better than those of UC-MSCs.

In this study, we further detected the effect of HS-pretreated UC-MSCs on macrophages, as macrophages are critical immune cells that reside in the lung to mediate adaptive and innate immunity [43, 44]. In addition, macrophages are also known to be a heterogeneous population of immune cells and are mainly divided into two subgroups according to their functions and biomarkers. M1 macrophages have a proinflammatory phenotype, secrete various kinds of proinflammatory cytokines and chemokines, and play an important role in antigen presentation; in contrast, M2 macrophages are immunoregulatory cells that secrete IL-10 and TGF-β. During ALI, the activation of macrophages by LPS disrupts the balance of the immune status, and this effect is closely associated with a superabundant inflammatory response and subsequent lung injury [9]. Thus, recent studies have demonstrated that affecting macrophage polarization, which inhibits abnormally activated macrophages and promotes a regulatory phenotype, could be a therapeutic approach for ALI [45, 46]. By collecting BALF and culture supernatants, our in vivo and in vitro studies not only demonstrated the effect of UC-MSCs on regulating the phenotypic polarization of macrophages but also revealed that HS pretreatment enhanced the immunosuppressive effects of UC-MSCs, which further notably reduced the proportion of activated macrophages and increased polarization toward an immunoregulatory phenotype.

Mechanistically, activating macrophages with LPS upregulated TLR4 expression subsequently stimulated its downstream signaling pathway, including NF-κB, c-jun N-terminal kinase, and p38, and finally activated the NLRP3 inflammasome to synthesize various kinds of proinflammatory cytokines, such as IL-1β and IL-18 [47]. The imbalance of anti-inflammatory and proinflammatory responses leads to the progression of ALI. Previous research found that melatonin alleviated ALI by inhibiting the NLRP3 inflammasome [48]. Yin et al. performed in vivo and in vitro experiments to demonstrate that isoflurane acted as a therapeutic approach to improve LPS-induced ALI by inhibiting NLRP3 inflammasome activation in alveolar macrophages [49]. MSCs also play an important role in modulating inflammasome activation. In an in vitro study, Joo et al. found that MSCs inhibited Caspase-1 activation and IL-1β secretion in activated macrophages by reducing the level of mitochondrial reactive oxygen species (ROS) [50]. In murine models of rheumatoid arthritis and Crohn’s disease, Won and Serena et al. showed that MSCs could block inflammasome activation to regulate the immune properties of macrophages [51, 52]. In addition, Liu found that MSC-derived exosomes also reduced inflammasome activation in macrophages to alleviate LPS/galactosamine-induced acute liver failure via their encapsulated microRNAs [53]. In the present study, our results from the in vivo studies suggested that UC-MSCs could notably inhibit NLRP3 inflammasome activation in lung tissues, and further in vitro studies revealed that MSCs could successfully block the expression of NLRP3, ASC, and cleaved-Caspase 1 in macrophage cell lines. In summary, we concluded MSCs may be utilized as a therapeutic approach to attenuate ALI by inhibiting NLRP3 inflammasome activation. Shi Yue et al. proved that heat shock transcription factor 1 (HSF1) activation promoted β-catenin expression, which led to NLRP3 inactivation and decreased I/R-induced liver injury. These authors indicated HSF1/β-catenin signaling is a novel regulator of innate immunity during liver inflammatory injury [54]. Whether HS-pretreated MSCs modulate macrophages has not been reported until now. Interestingly, we also observed, for the first time, that HS pretreatment not only promoted the effects of MSCs in lung protection but also enhanced the potential of MSCs to modulate the NLRP3 inflammasome in macrophages. Wang et al. proved that MSC-derived PGE2 inhibited TGF-β-activated kinase 1 (TAK1) signaling and NLRP3 inflammasome activation in liver macrophages to decrease the production of inflammatory cytokines [55]. Therefore, in this study, we measured the levels of cytokines secreted by MSCs to determine how HS pretreatment enhanced the therapeutic potential of MSCs. Our findings suggested that HS pretreatment resulted in a decrease in NLRP3 inflammasome formation and IL-1β secretion by macrophages via enhancing PGE2 release. Cell death is induced by the NLRP3 inflammasome, and this form of cell death is called pyroptosis. In the lung, airway macrophages are critical for maintaining the functionality of airways via the clearance of inhaled particles, cell debris, and infectious agents [56]. MSC death modulation appears to be a decisive biological effect that could explain a significant portion of the therapeutic effects of MSCs [57, 58]. Other researchers found that exposing activated THP-1 cells with NLRP3 or ASC knocked down to indium-tin-oxide nanoparticles resulted in cell death without cytolysis, with deficiency in IL-1β/TNF-α, and with features of apoptosis. Mesenchymal stem cells (MSCs) cocultured with macrophages inhibited both the inflammation and cell death induced by indium-tin-oxide nanoparticles [59]. As previous evidence from Fan et al. showed that HSP70 could upregulate COX-2 expression to promote inflammation, we finally detected the expression levels of members of a family of heat shock proteins that are substantially upregulated during stress conditions, including infection, oxidative stress, inflammation, and HS [35, 36]. As expected, HS pretreatment induced HSP70 expression in MSCs. Our data from knocking down HSP70 in conjunction with investigating the effects of HS-pretreated MSCs on NLRP3 inflammasome activation in macrophages further confirmed the role of HS pretreatment in enhancing the immunoregulatory effects of MSCs.

In conclusion, the present study revealed that HS pretreatment can be used to enhance the immunoregulatory capacity of MSCs and to improve ALI. It is well known that the expression of heat shock proteins is highly induced after HS pretreatment. Our findings of increased HSP70 expression in UC-MSCs indicate that a high level of HSP70 enhances PGE2 secretion by UC-MSCs, which inhibits NLRP3 inflammasome activation in macrophages. Therefore, HS pretreatment may be a promising strategy to enhance the therapeutic potential of MSCs in the treatment of ALI in the clinic.

## Supplementary Information

**Additional file 1.**

## Data Availability

The datasets used and/or analyzed during the current study are available from the corresponding author on reasonable request.

## Abbreviations

LPS: Lipopolysaccharide
ALI: Acute lung injury
MSCs: Mesenchymal stem cells
UC-MSCs: Umbilical cord-derived mesenchymal stem cells
HS: Heat shock
HSP70: Heat shock protein 70
BALF: Bronchoalveolar lavage fluid
ARDS: Acute respiratory distress syndrome
IRI: Ischemia/ reperfusion injury
Ang-1: Angiopoietin-1
IL-10: Interleukin-10
KGF: Keratinocyte growth factor
PGE2: Prostaglandin E2
PBS: Phosphate-buffered saline
PFA: Paraformaldehyde
qRT-PCR: Quantitative real-time reverse transcriptase-polymerase chain reaction
MSC-CM: MSC conditioned medium
ELISA: Enzyme-linked immunosorbent assay
RIPA: Radioimmunoprecipitation assay
EDTA: Ethylene diamine tetraacetic acid
SEM: Standard error of the mean
COXs: Cyclooxygenases
