# Supplementary material for: Heat shock preconditioning mesenchymal stem cells attenuate acute lung injury via reducing NLRP3 inflammasome activation in macrophages
Source: Stem Cell Res Ther. 2021 May 17;12:290. doi: 10.1186/s13287-021-02328-3 (PMC8127288; doi:10.1186/s13287-021-02328-3)
Supplement: Supplementary file 1 — Additional file 1. [file 13287_2021_2328_MOESM1_ESM.docx]

Supplemental Table 1. Primer sets used in this study. Related to Figure 3

| Name |  | Sequences (5'-3') |
| --- | --- | --- |
| GAPDH | Forward | AGAAGGCTGGGGCTCATTTG |
|  | Reverse | AGGGGCCATCCACAGTCTTC |
| IL-1β | Forward | GCCAGTGAAATGATGGCTTATT |
|  | Reverse | AGGAGCACTTCATCTGTTTAGG |
| IL-6 | Forward | CACTGGTCTTTTGGAGTTTGAG |
|  | Reverse | GGACTTTTGTACTCATCTGCAC |
| TNF-α | Forward | GAGGCCAAGCCCTGGTATG |
|  | Reverse | CGGGCCGATTGATCTCAGC |
|  | Reverse | AAAGGGGATACAGGTTTTTCCAC |

Supplemental Table 2. Primer sequences for the knockdown of HSP70, related to Figures 5and 6.

|  | Sequences (5'-3') |
| --- | --- |
| shHSP70 | GGCCAACAAGATCACCATC |
